# A Novel Factor Graph and Cubature Kalman Filter Integrated Algorithm for Single-Transponder-Aided Cooperative Localization

**DOI:** 10.3390/e23101244

**Published:** 2021-09-24

**Authors:** Wanlong Zhao, Huifeng Zhao, Deyue Zou, Lu Liu

**Affiliations:** 1 School of Information Science and Engineering, Harbin Institute of Technology, Weihai 264209, China; wlzhao@hit.edu.cn; 2 College of Underwater Acoustic Engineering, Harbin Engineering University, Harbin 150001, China; zhf@hrbeu.edu.cn; 3 School of Information and Communication Engineering, Dalian University of Technology, Dalian 116081, China

**Keywords:** cooperative localization, CKF, factor graph, single transponder

## Abstract

Cooperative localization (CL) of underwater multi-AUVs is vital for numerous underwater operations. Single-transponder-aided cooperative localization (STCL) is regarded as a promising scheme for multi-AUVs CL, benefiting from the fact that an accurate reference is adopted. To improve the positioning accuracy and robustness of STCL, a novel Factor Graph and Cubature Kalman Filter (FGCKF)-integrated algorithm is proposed in this paper. In the proposed FGCKF, historical information can be efficiently used in measurement updating to overcome uncertain observation environments, which greatly helps to improve the performance of filtering progress. Furthermore, Adaptive CKF, sum product, and Maximum Correntropy Criterion (MCC) methods are designed to deal with outliers of acoustic transmission delay, sound velocity, and motion velocity, respectively. Simulations and experiments are conducted, and it is verified that the proposed FGCKF algorithm can improve positioning accuracy and robustness greatly than traditional filtering methods.

## 1. Introduction

Multi-AUVs are widely used in underwater operations, which has become a significant trend for environment and oceanography research to improve operation efficiency and diversity concurrently [1]. Cooperative localization (CL) of multi-AUVs is an efficient technology in deep-sea areas, especially where Global Navigation Satellite System (GNSS) is unavailable due to the attenuation of wireless signal. Generally, navigation aid is necessary for CL systems to provide exact position information for revising system deviation. Autonomous Surface Vehicle (ASV) and surface buoy are common navigation aids [2]; however, these surface units are affected by ocean currents, as well as lack of crypticity. In this paper, a single transponder is adopted as the navigation aid [3], which is deployed and calibrated at the seafloor in advance.

One of the core problems is the positioning algorithm in CL. There have been many algorithms proposed to realize underwater multi-AUVs CL. Least square (LS) is a typical CL algorithm, including square-range LS with Gaussian variational message passing [4], LS combining rank-relaxed semidefinite programming and maximum likelihood estimation [5], and iterative LS estimators [6]. LS focuses on dealing with a multilateral positioning method, which is not suitable for single-transponder-aided CL environments. Some other methods have been studied; for example, an iterative greedy algorithm has been proposed for collaborating underwater vehicles tasks [7]. In absolute terms, Bayesian filters provide more ideas to cope with CL problem, and a great number of algorithms based on Bayesian have been explored, such as Moving Horizon Estimation (MHE) integrated with Extended Kalman Filter (EKF) [8], EKF with measurement rough estimation algorithm [9], passive-tracking based on EKF [10], EKF based belief propagation [11], Multi-Hypothesis Extended Kalman Filter (MHEKF) [12], Particle Filtering (PF) [13], and Optimal Bayesian Kalman filter [14]. Among these methods, Cubature Kalman Filter (CKF) has a specific advantage in nonlinear estimation problems [15] and is a promising scheme in CL. Though CKF performs well in nonlinear estimation accuracy, it lacks system robustness when applied in CL. Actually, filters can improve its adaptation for measurement outliers to improve algorithm robustness. Reference [16] considered measurement noise as a heavy-tailed mixture (HTM) distribution and a Normal-Gamma-inverse Wishart distribution. Student’s t-based EKF algorithm [17] and Expectation Maximization (EM) [18] show an improved robustness against outliers found in the process and measurement noises, respectively. There have been some adaptive methods proposed to maintain filters’ Gaussian feature. The authors [19] presented an Adaptive Extended Kalman Filter (AEKF) based on the EM method. An adaptive filter using a forgetting factor has been presented to calculate noise matrix [20]. A measurement information detection based on Adaptive Neuro Fuzzy Inference System (ANFIS) is designed in [21]. In view of the previous scholarly studies, an Adaptive Cubature Kalman Filter (ACKF) is proposed in this paper, aiming to improve the robustness of CL method.

One disadvantage is that only current measurement information is used in filter methods. Graph optimization theory can be an ideal integrated scheme for filter in CL, benefiting from its combined use of historical information. Factor Graph (FG) is a promising graph optimization method, and sum-product algorithm is the core supporting to deal with message passing [22,23]. There have been a wide variety of algorithms proposed related to sum-product, including artificial intelligence, signal processing, digital communications, and positioning and navigation. A factor-graph and sum-product-based cooperative positioning algorithm by local function estimation is proposed in the literature [24]. Sun et al. proposed a CL algorithm based on a hybrid topology architecture using relative measurement graph method [25]. In the literature [26], Gaussian parameterizations of messages are combined in the framework of factor graph. Factor graph can merge different available asynchronous sensor information efficiently and accurately [27] and can be readily modified by adding corresponding factor nodes and edges [28]. Considering the nonlinear filtering capacity of CKF and efficient utilization of historical information in distributed topology of factor graph, a Factor Graph and Cubature Kalman Filter (FGCKF)-integrated algorithm is proposed in this paper, which aims to improve the positioning accuracy and promote in single-transponder-aided Cooperative Localization (STCL) of underwater multi-AUVs operation.

In the proposed FGCKF in STCL, outliers of multidimensional information are considered, including acoustic transmission delay, sound velocity, and motion velocity, etc. ACKF is supposed to overcome outliers of transmission delay. As for the outliers of sound velocity, an estimating method for sum-product based Effective Sound Velocity (ESV) [29] is designed. Based on historical factor graph list, ESV can be updated with transmission delay simultaneously and can further be treated as a random variable participating in the filtering process in ACKF. Motion velocity of AUV can also contain outliers during long time operation in a deep-sea environment. Though current velocity is believed to revise accurate motion velocity by some scholars [30,31], the building and updating of the current velocity model is very difficult. Maximum Correntropy Criterion (MCC) has recently been successfully applied in robust regression and filtering [32] and active noise control [33]. In FGCKF, MCC is adopted to deal with the outlier problem of motion velocity.

The contributions of this paper are as follows. A factor graph and CKF-integrated algorithm is proposed in single-transponder-aided Cooperative Localization in underwater multi-AUVs operations. It aims not only to improve the positioning accuracy but also to enhance the robustness greatly in CL schemes. Furthermore, in the proposed FGCKF, an Adaptive CKF method is presented to focus on the adjustment of the measurement covariance matrix for acoustic transmission delay. ESV estimation based on a sum product algorithm is described to improve accuracy of ranging measurements. An MCC based reconstruction method is adopted to overcome the uncertainty problem of motion velocity.

The rest of this paper is organized as follows. Section 2 presents the framework of STCL. The proposed FGCKF is described in detail in Section 3. In Section 4, simulations and experiments are conducted. Finally, conclusions are given in Section 5.

## 2. Framework of STCL

Generally speaking, there are two modes of multi-AUVs CL technology, one is the master–slave mode, and the other is parallel mode. Benefiting from saving positioning equipment and navigation cost, the master–slave mode is attracting more and more attention. In master–slave mode, the main AUV, which can position itself with high-accuracy navigation equipment, is considered the master AUV. Other AUVs that reach their locations using the master AUV are named slave AUVs. Ranging and location information are transmitted by acoustic communication links between master and slave.

**Figure 1:**
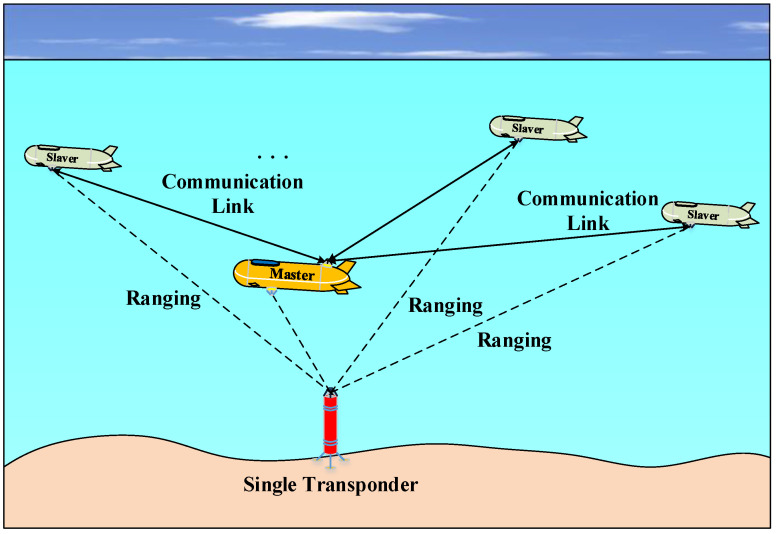
Illustration of framework of STCL.

In traditional master–slave CL, a master AUV, equipped with high-accuracy Inertial Navigation System (INS) and GNSS, needs to rise to sea surface to update global location periodically. Even if it avoids rising to the surface, a surface unit, such as an ASV or buoy, must be present as a navigation aid. However, when multi-AUVs operate in a deep sea environment, it is a time-consuming and energy-intensive process for the master to rise to the sea surface; meanwhile, it is hard to build steady communication link between surface unit and deep sea master. In this paper, a novel Single Transponder aid CL (STCL) scheme is designed. Unlike traditional the acoustic technology Long Base-line (LBL) positioning system [34], where at least three transponders need to be deployed and calibrated at the seabed or lakebed in advance, only one transponder is adopted in STCL. Although it is difficult to calculate accurate locations using one series of acoustic transmission delays, it can play an efficient supporting role to aid CL. As the water depths vary greatly in different environments, it will lead a significant error for ranging measuring, which will be solved by an ESV estimating method detailed in next Section. The framework of STCL is shown in Figure 1.

In this STCL framework, all multi-AUVs can achieve ranging information between AUV and a single transponder by periodic acoustic impulse response. The master AUV is equipped with high-accuracy INS, which can provide accurate motion-velocity information, and hence the master can be positioned, merging velocity information and single transponder ranging information precisely. However, taking the cost into consideration, slave AUVs are equipped with Doppler Velocity Log (DVL) instead of INS. The results is that only a rough motion velocity can be achieved even with certain outliers. This presents a prime challenge to STCL, namely how to position slave AUVs by making full use of master–slave communication link, master ranging, single transponder ranging, rough motion velocity, etc.

To solve the main problem in STCL, a factor graph and CKF integrated algorithm is proposed and shown in detail in the following section.

## 3. FGCKF in STCL

### 3.1. The Proposed FGCKF Algorithm

Considering the STCL as a discrete time system, we define a state vector at time *k* to describe the slave AUV dynamic state as

(1) $$x_{k}={\left[x_{k},y_{k},z_{k},v_{k},\theta_{k},v_{e,k}\right]}^{T}$$

where $x_{k},y_{k},z_{k}$ denote the AUVs three-dimensional coordinates, $v_{k}$ and $v_{e,k}$ present motion velocity and sound velocity, respectively. $\theta_{k}$ indicates heading direction. State equation and measurement equation are built as follows.

(2) $$x_{k}=f\left(x_{k-1}\right)+w_{k}$$

(3) $$z_{k}=h\left(x_{k}\right)+\delta_{k}$$

where f(·) and h(·) are nonlinear transition functions for state and measurement updating. $z_{k}$ represents the measurement vector. $w_{k}∼N\left(0,Q_{k}\right)$, $\delta_{k}∼N\left(0,R_{k}\right)$ indicate state noise and measurement noise, respectively. $Q_{k}$ and $R_{k}$ denote the noise covariances of $w_{k}$ and $\delta_{k}$.

In the framework of STCK, the state transition function and measurement transition function are as follows.

(4) $$f=\left\{\begin{array}{c} x_{k}=x_{k-1}+v_{k}\cdot t\cdot \cos\theta_{k}\\ y_{k}=y_{k-1}+v_{k}\cdot t\cdot \sin\theta_{k}\\ z_{k}=z_{k}\\ v_{k}=v_{k-1}+w_{k}^{v}\\ \theta_{k}=\theta_{k-1}+w_{k}^{\theta }\\ v_{e,k}=g\left(z_{k}\right); \end{array}\right.$$

(5) $$h=\left\{\begin{array}{c} \tau_{b,k}=\frac{\sqrt{{\left(x_{k}-x_{b}\right)}^{2}+{\left(y_{k}-y_{b}\right)}^{2}+{\left(z_{k}-z_{b}\right)}^{2}}}{v_{e,k}}\\ \tau_{l,k}=\frac{\sqrt{{\left(x_{k}-x_{l,k}\right)}^{2}+{\left(y_{k}-y_{l,k}\right)}^{2}+{\left(z_{k}-z_{l,k}\right)}^{2}}}{v_{e,k}} \end{array}\right.$$

where *t* is time interval. $w_{k}^{v}$ and $w_{k}^{\theta }$ represent process noise of *v* and θ, respectively. g(·) represents the relationship between sound velocity and depth. $\tau_{b,k}$ presents transmission delay between slave and single transponder, $\tau_{l,k}$ presents transmission delay between slave and master. $\left(x_{b},y_{b},z_{b}\right)$ is single transponder location, and $\left(x_{l,k},y_{l,k},z_{l,k}\right)$ is master location.

The model of Kalman filter can be written as conditional probability density function $f(x_{1},\cdots ,x_{k}|z_{1},\cdots ,z_{k})$, in which $x_{k}$ is determined by $x_{k-1}$ and $z_{k}$, satisfying hidden Markov model condition. Further the function can be defined as:

(6) $$f\left(x_{1},\cdots ,x_{k}|z_{1},\cdots ,z_{k}\right)=\prod_{i=1}^{k}f\left(x_{i}|x_{i-1}\right)f\left(z_{i}|x_{i}\right)$$

The state and measurement variables are assumed to satisfy Gaussian distribution [35]; hence the state prediction Probability Density Function and measurement transition Probability Density Function are described as follows.

(7) $$\begin{array}{c} f\left(x_{i}|x_{i-1}\right)=N\left(f\left(x_{i-1}\right),Q_{i}\right) \end{array}$$

(8) $$\begin{array}{c} f\left(z_{i}|x_{i}\right)=N\left(h\left(x_{i}\right),R_{i}\right) \end{array}$$

To make a better state information fusing, a factor graph is employed in STCL. A factor graph (FG) is a kind of bi-directional graph for information transfer that can cope with complex multivariate global functions. Moreover, FG is strong in plug-and-play and asynchronous fusion, which is valuable applying in STCL.

There are two category nodes in FG, including variable node and function node. More specifically, it graphically represents the factorization structure of global function as the product of local functions. Messages are transmitted following the sum-product in FG. At time slot *k*, the message from state node $x_{k-1}$ and measurement node $z_{k}$ are fused on node $x_{k}$.

(9) $$\begin{array}{cc} \mu_{k|k-1}\left(x_{k-1}\right) & =\int \mu_{k-1|k-1}\left(x_{k-1}\right)f\left(x_{k}|x_{k-1}\right)dx_{k-1}\\ \; & =\int \mu_{k-1|k-1}\left(x_{k-1}\right)N\left(f\left(x_{k-1}\right),Q_{k}\right)\\ \; & \propto N(m_{k|k-1},P_{k|k-1}) \end{array}$$

(10) $$\begin{array}{cc} \mu_{k|k}\left(x_{k}\right) & =\mu_{k|k-1}\left(x_{k}\right)f\left(z_{k}|x_{k}\right)\\ \; & =N\left(m_{k|k-1},P_{k|k-1}\right)N\left(h\left(x_{k}\right),R_{k}\right)\\ \; & \propto N(m_{k|k},P_{k|k}) \end{array}$$

where $m_{k|k}$ is equal to $x_{k}$.

Furthermore, based on Equations (9) and (10), the Cubature Kalman Filter (CKF) is adopted to finish updating of state information.

CKF depends on transforming a set of cubature points using state and measurement models to realize nonlinear estimation. The key to propagate the cubature points is the Cholesky decomposition of error covariance. The following equations show how to use cubature points to calculate the mean and variance of the state.

(11) $$P_{k-1}=S_{k-1}S_{k-1}^{T}$$

(12) $$x_{i,k-1}=S_{k-1}\xi_{i}+x_{k-1}\;i=1,\cdots ,2n$$

(13) $$x_{i,k|k-1}=f\left(x_{i,k-1}\right)$$

(14) $$x_{k|k-1}=\frac{1}{2n}\sum_{i=1}^{2n}x_{i,k|k-1}$$

(15) $$P_{k|k-1}=\frac{1}{2n}\sum_{i=1}^{2n}x_{i,k|k-1}x_{i,k|k-1}^{T}-x_{k|k-1}x_{k|k-1}^{T}+Q_{k}$$

where $P_{k-1}$ and $P_{k|k-1}$ denote error covariance and predicted error covariance at time k−1, respectively. $S_{k-1}$ presents Cholesky decomposition result. $x_{i,k|k-1}$ indicates propagating cubature points. $x_{k|k-1}$ is the predicted state. The set of points $\xi_{i}$ adopts $\sqrt{n}{\left[1\right]}_{i}$.

Cubature points are also propagated to accomplish the estimation summarized as follows.

(16) $$P_{k|k-1}=S_{k|k-1}S_{k|k-1}^{T}$$

(17) $$x_{i,k|k-1}=S_{k|k-1}\xi_{i}+x_{k|k-1}\;i=1,\cdots ,2n$$

(18) $$z_{i,k|k-1}=h\left(x_{i,k|k-1}\right)$$

(19) $$z_{k|k-1}=\frac{1}{2n}\sum_{i=1}^{2n}z_{i,k|k-1}$$

(20) $$P_{z,k|k-1}=\frac{1}{2n}\sum_{i=1}^{2n}z_{i,k|k-1}z_{i,k|k-1}^{T}-z_{k|k-1}z_{k|k-1}^{T}+R_{k}$$

(21) $$P_{xz,k|k-1}=\frac{1}{2n}\sum_{i=1}^{2n}x_{i,k|k-1}z_{i,k|k-1}^{T}-x_{k|k-1}z_{k|k-1}^{T}$$

(22) $$K_{k}=P_{xz,k|k-1}P_{z,k|k-1}^{-1}$$

(23) $$x_{k}=x_{k|k-1}+K_{k}\left(z_{k}-z_{k|k-1}\right)$$

(24) $$P_{k}=P_{k|k-1}-K_{k}P_{z,k|k-1}K_{k}^{T}$$

where $z_{i,k|k-1}$ is cubature points of observation, $z_{k|k-1}$ is predicted measurement vector, $P_{z}$ is autocorrelation covariance matrix, $P_{xz}$ is cross-correlation covariance matrix, and K is Kalman gain. Based on Equations (16)–(24), the state vector and error covariance are updated. The fusion result is achieved by certain iterations.

CKF utilizes cubature points to update the state, which makes the system mean and variance reach the third-order accuracy. Compared with EKF in first-order accuracy, it can improve the updating accuracy greatly.

**Figure 2:**
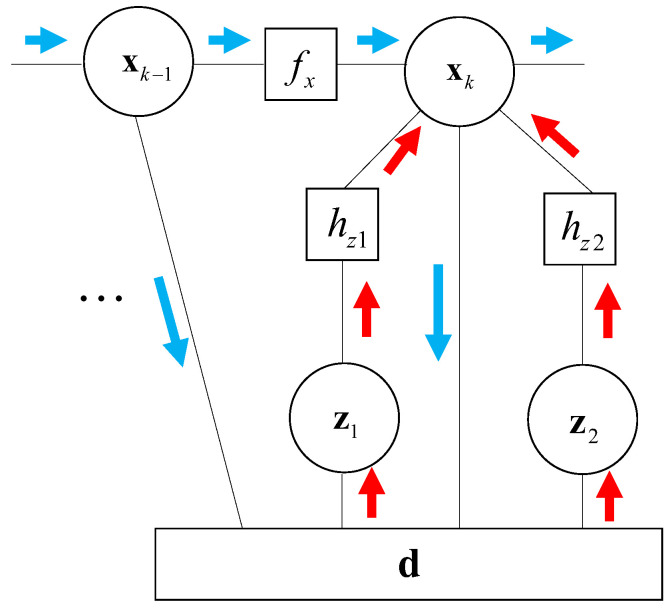
Message passing mode of FGCKF.

As mentioned above, the state evolves compared to the former state at time k−1 and current measurement at time *k*. However, there is a large amount of historical measuring information ignored in the filtering process. It is known that historical information is efficiently used by message passing in the FG framework. Therefore, the message passing mode in FGCKF is designed, and its procedure is shown in Figure 2.

In Figure 2, $x_{k}$, $x_{k-1}$ represent state nodes in time *k* and time k−1, respectively. $z_{1}$ indicates measuring transmission delay vector between slave and transponder, and $z_{2}$ indicates measuring transmission delay vector between slave and master. $f_{x}$ denotes state prediction node. $h_{z1}$ and $h_{z2}$ present observation functions of $z_{1}$ and $z_{2}$. d is set to deal with observation residuals. The blue arrows represent the messages sent out by state node x, and the blue arrows represent the messages sent into state node x.

In the message passing of transmission delay, there exists certain noises of measurement error. Let τ present transmission delay, and $\tau^{b}$, $\tau^{l}$ denote transmission delay from single transponder and master AUV, respectively. Besides the direct measurement variable $\tau_{m}$, transmission delay can also be calculated in the former process of CKF, expressed as $\tau_{c}$.

During every update step in CKF, a real-time position of AUV $\left(X_{k},Y_{k},Z_{k}\right)$ is obtained. Assuming location of transponder is $\left(X_{b},Y_{b},Z_{b}\right)$, location of master AUV is $\left(X_{l},Y_{l},Z_{l}\right)$, and then $\tau_{c}$ in time *k* is expressed as

(25) $$\begin{array}{c} \tau_{c,k}^{b}=\frac{\sqrt{{\left(X_{k}-X_{b}\right)}^{2}+{\left(Y_{k}-Y_{b}\right)}^{2}+{\left(Z_{k}-Z_{b}\right)}^{2}}}{v_{e}}\\ \tau_{c,k}^{l}=\frac{\sqrt{{\left(X_{k}-X_{l,k}\right)}^{2}+{\left(Y_{k}-Y_{l,k}\right)}^{2}+{\left(Z_{k}-Z_{l,k}\right)}^{2}}}{v_{e}} \end{array}$$

where $v_{e}$ indicates sound velocity. A delay error value γ is designed based on the difference between $\tau_{c}$ and $\tau_{m}$,

(26) $$\gamma_{k}=\left|\tau_{m,k}-\tau_{c,k}\right|,$$

Then the delay error vector $\gamma =[\gamma_{1},\cdots ,\gamma_{k-1},\gamma_{k}]$ is built, whose variance can represent the measurement error magnitude. We use $\gamma_{b}$ to represent measurement error value related to transponder and $\gamma_{l}$ to represent measurement error value related to master AUV. The mean and variance of $\gamma_{b}$ and $\gamma_{l}$ can be calculated at time *k* as follows.

(27) $$\bar{\gamma }=\frac{1}{k}\sum_{i=1}^{k}\gamma_{i}$$

(28) $$\sigma_{\gamma }=\sqrt{\frac{1}{k-1}\sum_{i=1}^{k}{\left(\gamma_{i}-\bar{\gamma }\right)}^{2}}$$

Two sets of mean and variance of error value γ can be achieved based on $\tau^{b}$ and $\tau^{l}$, namely ${\bar{\gamma }}_{b}$, $\sigma_{\gamma ,b}$ and ${\bar{\gamma }}_{l}$, $\sigma_{\gamma ,l}$. Based on the sum product rule, this information is fused using the Gaussian product rule, which is shown as follows.

(29) $$\frac{1}{\Sigma_{\gamma }^{2}}=\frac{1}{\sigma_{\gamma ,b}^{2}}+\frac{1}{\sigma_{\gamma ,l}^{2}}$$

Based on the fused variance $\Sigma_{\gamma }^{2}$, vector d is designed to adjust measurement value. The transmission delay $\tau_{k+1}$ in time k+1 is defined as

(30) $$\begin{array}{c} \tau_{k+1}=\tau_{m,k+1}-d_{k}\\ d_{k}=\left\{\begin{array}{cc} \Sigma_{\gamma } & \;k>k_{c}\\ 0 & \;k\leq k_{c} \end{array}\right. \end{array}$$

where $k_{c}$ is the adjustment threshold, while $k\leq k_{c}$, $\Sigma_{\gamma }$ is considered not valuable enough to be the adjustment pattern of τ.

### 3.2. Adaptive CKF

As mentioned in Section 3.1, measurement noise matrix R of acoustic transmission delay influences Kalman gain, which determines the proportion of trust between observation variable and state transferring variable. When there exists outliers of transmission delay, the calculation of Kalman gain will deteriorate. In this section, an Adaptive CKF (ACKF) is designed to resist delay outliers by renewing measurement noise matrix R. The details are shown in the following.

Firstly, an adaptive scaling factor α is introduced. The measurement noise matrix $R_{k}$ in time *k* is expressed as

(31) $$R_{k}=R_{k-1}/\alpha$$

By adaptive changing scaling factor α, it is enabled to adjust the system noise covariance matrix for online tuning of the predicted state covariance. When meeting the high precision measurement, the weight of measurement in the state estimation is enhanced. Otherwise, the weight of low precision measurement is decreased. A screening model is established to classify the observation residual, which is analogous to the IGG III equivalent weight function model [36].

(32) $$\alpha =\left\{\begin{array}{cc} 1 & \;s_{v}\leq \beta_{0}\\ \frac{\beta_{0}}{s_{v}}\left(\frac{\beta_{1}-s_{v}}{\beta_{1}-\beta_{0}}\right) & \;\beta_{0}<s_{v}<\beta_{1}\\ 10^{-10} & \;s_{v}\geq \beta_{1}, \end{array}\right.$$

where $\beta_{0}$ and $\beta_{1}$ are screening standard parameters and $s_{v}$ is normalized residual. Let $\epsilon_{k}=z_{k}-h\left(x_{k|k-1}\right)$ be the measurement residual; $s_{v}$ is expressed as

(33) $$s_{v}=\frac{\epsilon_{k}}{\sigma_{k}},$$

where $\sigma_{k}$ is variance factor obtained by $median(s_{i})/0.6745$. $s_{i}$ is the set of all residual values.

Variance factor is mainly used to standardize residual, which is related to the detection and discrimination of errors. Moreover, $\beta_{0}$ and $\beta_{1}$ are usually regarded as constant experienced values. Based on traditional practical experience, $\beta_{0}$ adopts 1.5–2.0, and $\beta_{1}$ adopts 3.0–8.5. Considering complex underwater environment, we set $\beta_{1}$ to be alterable as follows:

(34) $$\beta_{1}=max(|{\hat{s}}_{i}\left|\right)/median\left(s_{i}\right)/0.6745,$$

where ${\hat{s}}_{i}$ indicates the set of residual values without outliers.

When there are more samples participated into the adaptive changing process, the alternative $\beta_{1}$ will be more suitable for enhancing accuracy of scaling factor.

### 3.3. Sum-Product Based ESV

Sound velocity (SV) is a vital element for estimation of acoustic range. However, SV changes in various ways following the changing environments. Achieving an Effective Sound Velocity (ESV) is an essential part of FGCKF in STCL. In this section, a sum-product-based ESV calculating method is proposed.

Assuming that ESV is $v_{e,k}$ at time slot *k*, the posterior probability of state variable related to ESV can be given by

(35) $$\begin{array}{c} p\left(x_{k},v_{e,k}|z_{k}\right)\propto \left(x_{k}|x_{k-1},v_{e,k-1}\right)p\left(v_{e,k}|v_{e,k-1}\right)p\left(z_{k}|x_{k-1}\right) \end{array}$$

In the factor graph of FGCKF, the prediction message is shown as

(36) $$\mu \left(x_{k}|x_{k-1}\right)=f\left(x_{k}|x_{k-1},v_{e,k-1}\right)\mu \left(x_{k-1}\right)\mu \left(v_{e,k-1}\right)$$

The message of current state can be updated by

(37) $$\mu \left(x_{k}\right)=f\left(z_{k}|x_{k-1}\right)\mu \left(x_{k}|x_{k-1}\right)$$

and the message of current ESV is expressed as $\mu (v_{e,k}^{-})$.

(38) $$\mu \left(v_{e,k}^{-}\right)=g\left(v_{e,k}^{-}|x_{k}\right)\mu \left(x_{k}\right)$$

Function node *g* is defined as

(39) $$g\left(v_{e,k}|x_{k}\right)=\frac{\|x_{k}-x_{b}\|}{\tau_{k}}$$

where $x_{b}$ presents location state of single transponder. Based on principle of the sum-product algorithm, the message of current ESV is updated further.

(40) $$\mu \left(v_{e,k}\right)=\mu \left(v_{e,k-1}\right)\mu \left(v_{e,k}^{-}\right)$$

**Figure 3:**
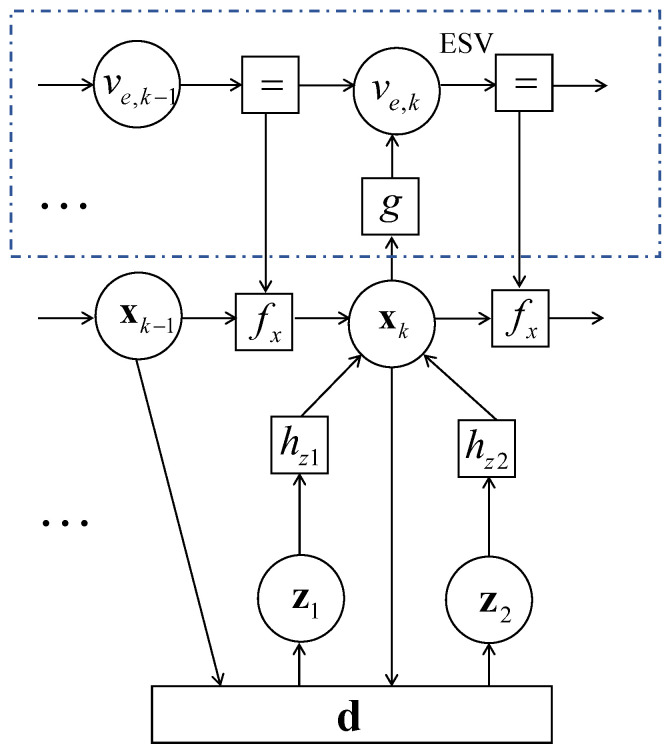
Sum-product based ESV in FGCKF.

Figure 3 gives the existing method of sum-product-based ESV in the FGCKF procedure. Benefiting from the continuous renewal of ESV, the accuracy of ranging estimation can be improved greatly.

### 3.4. MCC-Based Velocity Uncertainty

Motion velocity is significant information in slave localization in STCL. However, motion velocity cannot be measured very accurately using DVL, even with many outliers, which we call Velocity Uncertainty (VU). In this section, a Maximum Correntropy Criterion (MCC) method is adopted to solve the VU problem.

Correntropy is a generalized similarity measure between two random variables. V(x,y) represents correntropy between variable x and y, defined as

(41) $$V\left(x,y\right)=\Xi \left[\kappa \left(x,y\right)\right]=\int \kappa \left(x,y\right)dF_{xy}\left(x,y\right),$$

where Ξ denotes the expectation operator, $F_{xy}\left(x,y\right)$ is joint distribution function, and κ denotes a shift-variant kernel. In this paper, the kernel function adopts a Gaussian kernel for the noise is assumed as Gaussian random noise, shown as

(42) $$\kappa \left(x,y\right)=G_{\sigma }\left(x-y\right)=G_{\sigma }\left(e\right)=\frac{1}{\sqrt{2\pi }\sigma }\exp\left(-\frac{e^{2}}{2\sigma^{2}}\right),$$

where σ>0 is the kernel bandwidth.

The first step is to revise the velocity. The model can be expressed by the following equation,

(43) $$v_{k}^{-}={\hat{v}}_{k-1}+\eta$$

where η is Gaussian noise, which is subjected to N(0,Φ), $v_{k}^{-}$ is the prediction of velocity, and ${\hat{v}}_{k-1}$ represents the optimal estimate of velocity. Here the maximum correntropy is used as the cost function.

(44) $$J=G_{\sigma }\left(\right\|v_{k}-v_{k}^{-}\left\|\right)+G_{\sigma }\left(\right\|v_{k}-v_{k}^{m}\left\|\right)$$

where *J* represents correntropy of the velocity, $v_{k}^{m}$ is measured velocity which is obtained by DVL. Because of great VU, previous state and measurement are both used for velocity estimation. The optimal velocity is the one that makes the correntropy *J* reaches maximum. Furthermore, the partial derivatives is used for the optimal solution. The optimal $\hat{v}$ satisfies

(45) $$\begin{array}{cc} \frac{\partial J}{\partial v_{k}} & =\frac{\partial (G_{\sigma }\left(\right\|v_{k}-v_{k}^{-}\left\|\right)+G_{\sigma }\left(\right\|v_{k}-v_{k}^{m}\left\|)\right)}{\partial v_{k}}\\ \; & =\frac{\partial (\frac{1}{\sqrt{2\pi }\sigma }\left(\exp\left(-\frac{{\left(v_{k}-v_{k}^{-}\right)}^{2}}{2\sigma^{2}}\right)+\exp\left(-\frac{{\left(v_{k}-v_{k}^{m}\right)}^{2}}{2\sigma^{2}}\right)\right))}{\partial v_{k}}\\ \; & =0 \end{array}$$

(46) $$\begin{array}{c} {\hat{v}}_{k}=v_{k}^{-}+\left(v_{k}^{m}-v_{k}^{-}\right)\exp\left(-\frac{{\left(v_{k}^{-}-v_{k}^{m}\right)}^{2}}{2\sigma^{2}}\right)/\exp\left(-\frac{{\left(v_{k}^{-}-v_{k}^{-}\right)}^{2}}{2\sigma^{2}}\right)\\ \;=\;v_{k}^{-}+\left(v_{k}^{m}-v_{k}^{-}\right)\exp\left(-\frac{{\left(v_{k}^{-}-v_{k}^{m}\right)}^{2}}{2\sigma^{2}}\right) \end{array}$$

The outliers of motion velocity can be restrained efficiently using the MCC-based method.

### 3.5. Flow-Chart of FGCKF

By combining Adaptive CKF, Sum-product based ESV, MCC-based Velocity Uncertainty together, the proposed FGCKF algorithm is extended to be more complete and robust. The algorithm flowchart of FGCKF is summarized in Algorithm 1.
**Algorithm 1** Flowchart of proposed FGCKF in STCL.**Imput:** The initial state $x_{0}$ and $P_{0}$, time delay $\tau_{b,1:k}$ and $\tau_{l,1:k}$ and position of transponder and leader $\left(x_{b},y_{b},z_{b}\right)$ and ${\left(x_{l},y_{l},z_{l}\right)}_{1:k}$**Output:** The estimation $x_{1:k}$ and $P_{1:k}$ 1: **for**
*i* = 1:*k*
**do** 2: Calculate the velocity in Equation (46) 3: Calculate prediction state using Equations (11)–(15) 4: Revise measurement using Equation (30) 5: Calculate measurement noise covariance in Equations (31)–(34) 6: Calculate update state by Equations (16)–(24) 7: Estimate sound velocity using Equations (39) and (40) 8: Calculate measurement residual in Equations (25)–(29) 9: **end for**10: **return**
$x_{1:k}$ and $P_{1:k}$

## 4. Simulations and Experiments

In this section, both simulations and field experiments are conducted to verify the performance of the proposed scheme.

### 4.1. Simulation Results

We developed a cooperative localization simulation platform in Matlab, in which a single transponder is fixed in a position, and a master AUV and a slave AUV are deployed in this simulation scenes and move with virtual velocity and heading information. The sampling rates of AUVs and transponder are both set as 1 Hz. The simulations are all modeled under three dimension circumstance, and positioning error is calculated by $Error=\sqrt{{\left(x-x_{r}\right)}^{2}+{\left(y-y_{r}\right)}^{2}+{\left(z-z_{r}\right)}^{2}}$. Since the depth information *z* can be measured accurately by pressure sensor in actual operation, simulations focus principally on horizontal coordinates x,y. The simulations are conducted in three parts, namely performance analysis of ACKF, performance analysis of ESV, and performance analysis of integrated FGCKF.

#### 4.1.1. Performance Analysis of ACKF

**Figure 4:**
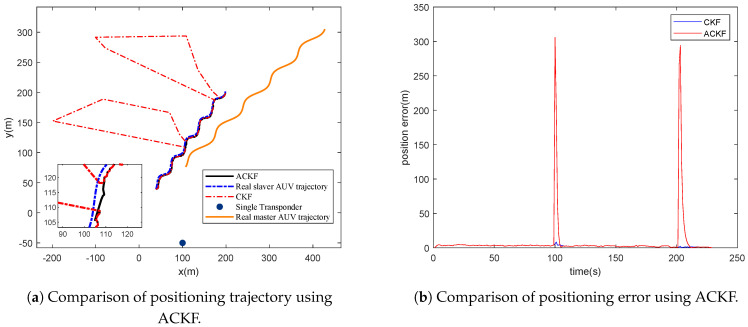
Performance comparison of ACKF and CKF with great outliers of transmission delay.

The first simulation is done to evaluate the robustness of ACKF. The sound velocity is set to be a constant as 1500 m/s. The single transponder is located at (100, −50, −100). The master slave moves with known trajectory coordinates. The positioned slave trajectory is compared with the preplanned trajectory (real trajectory) to calculate positioning error. In the ranging estimation, two parts of big noise are added artificially to represent the outliers of the acoustic transmission delay. Figure 4 gives the comparison of positioning trajectory and positioning error of ACKF and CKF, respectively.

From Figure 4a,b, it can be seen that ACKF performs better than CKF when facing unknown measurement noise. ACKF performs well in meeting the problem of measurement outliers, which indicates that ACKF improves the algorithm robustness significantly.

#### 4.1.2. Performance Analysis of ESV

**Figure 5:**
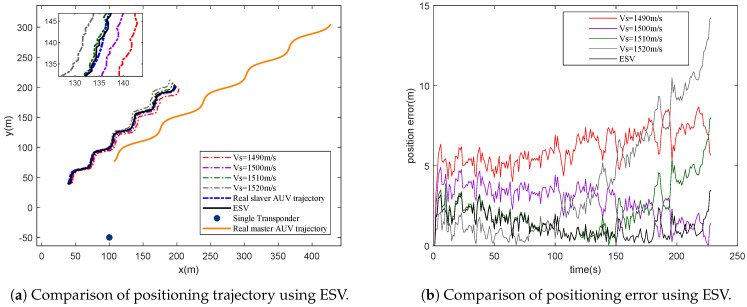
Performance comparison of various constant sound velocity and ESV.

Normally, sound velocity is set as a constant such as 1500 m/s. However, sound velocity varies, especially with changing depth, which will influence CL greatly. In this simulation, we use a sound velocity profile measured in the South China Sea to simulate different sound velocity at different depths, and four different sound velocities are chosen to compare with the proposed sum-product-based ESV. The basic simulation condition is the same as Performance Analysis of ACKF. The comparison results of various constant sound velocities and ESVs can be seen in Figure 5. It is clear that though the position error of constant sound velocity is sometimes lower than ESV, ESV performs much better in general. As a result, the CL benefits from the ESV, and the positioning accuracy is obviously improved. This encourages us to further investigate the features of ESV in the future.

#### 4.1.3. Performance Analysis of Integrated FGCKF

**Figure 6:**
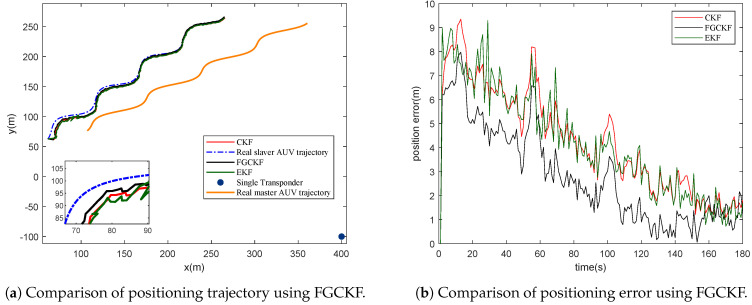
Performance comparison of proposed FGCKF, CKF and EKF.

In this section, the performance of the proposed integrated FGCKF is verified by comparing it with EKF and CKF. A single transponder is deployed at (400, −100, −100). Figure 6 shows the simulation result of FGCKF. From Figure 6a, it can be seen that the positioning trajectory of the proposed FGCKF is much closer to the real trajectory than CKF and EKF. Figure 6b shows that the average positioning error of FGCKF is the smallest among three methods. From this simulation, it can be concluded that the proposed FGCKF algorithm can efficiently improve positioning accuracy of STCL.

**Figure 7:**
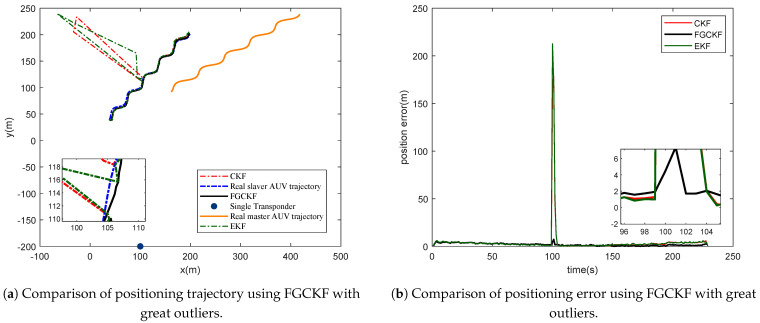
Performance comparison of proposed FGCKF, CKF and EKF with great outliers.

Furthermore, to evaluate the robustness of the proposed method, outliers of measurements, including transmission delay and motion velocity, are joined in this simulation. The outliers related simulation result is shown in Figure 7. It is clear that the positioning trajectory and positioning error of EKF and CKF make mutations when measurements meet outliers. Fortunately, the proposed FGCKF performs much better with obvious mutations with no matter positioning trajectory or positioning errors. From this simulation, it can be concluded that the proposed FGCKF improves robustness of STCL effectively.

**Figure 8:**
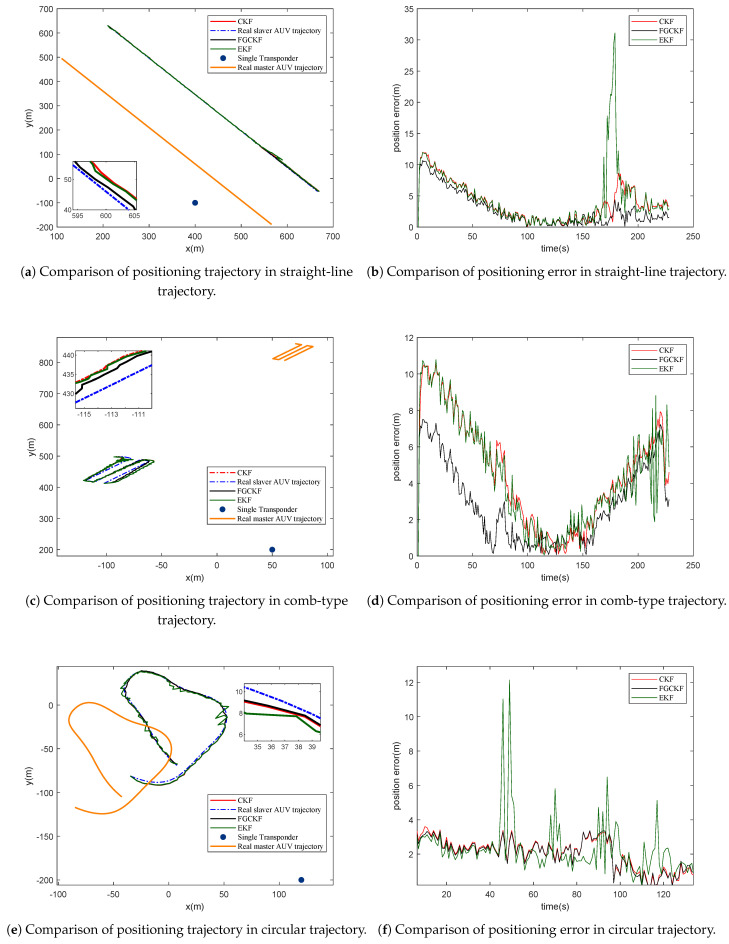
Performance comparison of proposed FGCKF, CKF, and EKF in different types of motion trajectory.

Besides the wave-like trajectory used in former simulations, three typical motion trajectories are considered, including straight-line trajectory, comb-type trajectory, and circular trajectory. Figure 8 describes the performance of FGCKF, CKF, and EKF with different motion trajectories. It can be seen that for strong linearity trajectories, including straight-line trajectory and comb-type trajectory, there is no obvious difference between CKF and EKF, and FGCKF performs much better than others. For non-linearity trajectories, including wave-like trajectory and circular trajectory, EKF performs worst, FGCKF and CKF perform better than EKF, and FGCKF is better than CKF.

Based on the above simulation results, it can be concluded that the proposed FGCKF can not only improve positioning accuracy but also effectively enhance robustness of STCL.

### 4.2. Field Data

In this section, the performance of FGCKF is verified by field data, which were collected using a lake experiment. The single transponder was deployed in the bottom of lake at (−65.7, −98.4, −64.5) in a local coordinate system. Furthermore, two AUVs were employed, where one served as the master and the other one as the slave. Both master and slave are equipped with acoustic transmitter and receiver, which help to realize ranging measuring between AUVs and transponder. The time interval of acoustic transmission delay gathering is 2 s. The slave AUV moved in a circular shape to imitate actual operations. Moreover, slave and master were both equipped with GPS to collect the true positions. The slave was equipped with a DVL to measure motion velocity information and a compass for heading information. Two different tests were conducted, including motionless master AUV and moving master AUV. On the condition of moving master AUV, it moved in a straight line at a constant velocity.

**Figure 9:**
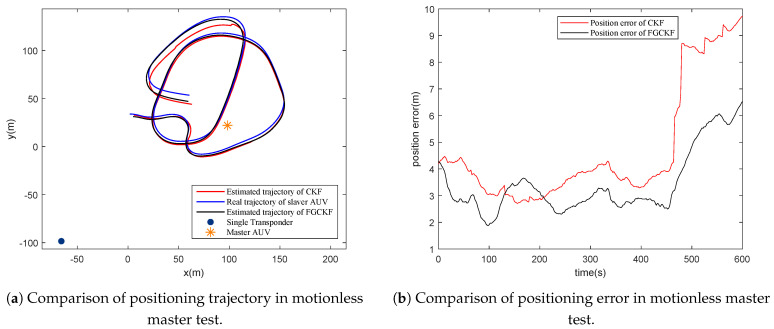
Performance analysis of proposed FGCKF with field data in motionless master test.

**Figure 10:**
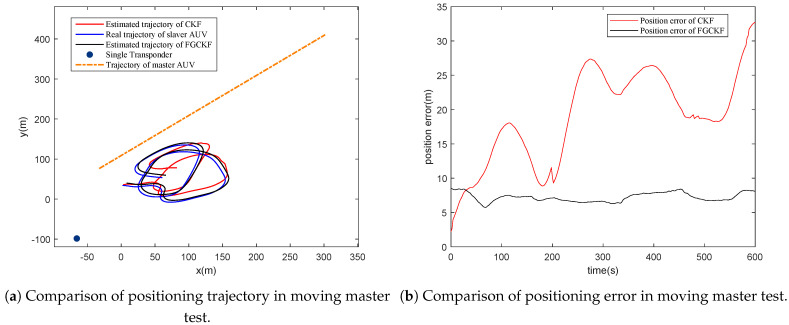
Performance analysis of proposed FGCKF with field data in moving master test.

The test results are shown in Figure 9 and Figure 10. From the tests, it can be seen that FGCKF and CKF maintain low errors most of the time, while FGCKF has lower errors than CKF. In particular, when encountering sharp measurement noise, CKF produces larger position errors, and FGCKF maintains positioning results stable, benefiting from its robustness.

From both simulation and experimental results, it can be seen that the proposed FGCKF can ensure accuracy and robustness concurrently in STCL.

## 5. Conclusions

In this paper, a novel factor graph and cubature Kalman filter-integrated algorithm is proposed for a single-transponder-aided cooperative localization underwater framework in master–slave AUV mode. In the proposed FGCKF, an adaptive CKF is presented in which adaptive measurement noise matrix is designed to overcome outliers of acoustic transmission delay. Traditional static sound velocity is replaced by sum-product-based ESV to make full use of historical sound velocity information in a factor graph. The outliers of the rough motion velocity measured by DVL in the slave AUV are restrained by the MCC method. Both simulation and experiment were conducted to verify the efficient performance of the proposed FGCKF, which lays a good foundation for future study of the positioning accuracy and robustness of FGCKF in STCL environments.

## Data Availability

Not applicable.
